# A family based tailored counselling to increase non-exercise physical activity in adults with a sedentary job and physical activity in their young children: design and methods of a year-long randomized controlled trial

**DOI:** 10.1186/1471-2458-11-944

**Published:** 2011-12-20

**Authors:** Taija Finni, Arja Sääkslahti, Arto Laukkanen, Arto Pesola, Sarianna Sipilä

**Affiliations:** 1 Neuromuscular Research Center, Department of Biology of Physical Activity, University of Jyväskylä, Jyväskylä, Finland; 2 Department of Physical Education, University of Jyväskylä, Jyväskylä, Finland; 3 Gerontology Research Centre, Department of Health Sciences, University of Jyväskylä, Jyväskylä, Finland

## Abstract

**Background:**

Epidemiological evidence suggests that decrease in sedentary behaviour is beneficial for health. This family based randomized controlled trial examines whether face-to-face delivered counselling is effective in reducing sedentary time and improving health in adults and increasing moderate-to-vigorous activities in children.

**Methods:**

The families are randomized after balancing socioeconomic and environmental factors in the Jyväskylä region, Finland. Inclusion criteria are: healthy men and women with children 3-8 years old, and having an occupation where they self-reportedly sit more than 50% of their work time and children in all-day day-care in kindergarten or in the first grade in primary school. Exclusion criteria are: body mass index > 35 kg/m^2^, self-reported chronic, long-term diseases, families with pregnant mother at baseline and children with disorders delaying motor development.

From both adults and children accelerometer data is collected five times a year in one week periods. In addition, fasting blood samples for whole blood count and serum metabonomics, and diurnal heart rate variability for 3 days are assessed at baseline, 3, 6, 9, and 12 months follow-up from adults. Quadriceps and hamstring muscle activities providing detailed information on muscle inactivity will be used to realize the maximum potential effect of the intervention. Fundamental motor skills from children and body composition from adults will be measured at baseline, and at 6 and 12 months follow-up. Questionnaires of family-influence-model, health and physical activity, and dietary records are assessed. After the baseline measurements the intervention group will receive tailored counselling targeted to decrease sitting time by focusing on commute and work time. The counselling regarding leisure time is especially targeted to encourage toward family physical activities such as visiting playgrounds and non-built environments, where children can get diversified stimulation for play and practice fundamental of motor skills. The counselling will be reinforced during the first 6 months followed by a 6-month maintenance period.

**Discussion:**

If shown to be effective, this unique family based intervention to improve lifestyle behaviours in both adults and children can provide translational model for community use. This study can also provide knowledge whether the lifestyle changes are transformed into relevant biomarkers and self-reported health.

**Trial registration number:**

ISRCTN: ISRCTN28668090

## Background

Parents of young children typically report low level of physical activity and many can be classified as inactive [1]. At the same time, parents face multidimensional challenges to support physical activity in their children [2]. While the physical activity guidelines stress the importance of moderate-to-vigorous exercises in both children and adults, evidence is merging that even small amounts of physical activity can have health-related benefits. One recent report shows that people who exercise 15 min more/day have 14% lower risk of all-cause mortality [3]. Conversely, every hour of TV viewing adds all-cause mortality by 11% [4]. Further, a physically active lifestyle has been shown to reduce genetic predisposition to obesity by 40%, and thus also the risk for obesity-related diseases [5].

Recently, daily inactivity time has been shown to be independent from purposeful physical activity [6]. Therefore, not only adding physical activity but avoiding inactivity has an important role in healthy lifestyle. Epidemiological studies have shown that sedentary time, independent from exercise, predicts all-cause mortality [7], obesity and type 2 diabetes [8,9], abnormal glucose metabolism [10,11], the metabolic syndrome [12,13] and cardiovascular disease [14]. In addition to the effects of total sedentary time, the pattern in which it is accumulated may be also important. Healy et al. [15] showed that total number of breaks in sedentary time was associated with lower waist circumference, triglycerides, and 2-h plasma glucose, independent of total sedentary time. These authors suggested that breaking prolonged periods of sitting might be a valuable addition to the public health recommendations. Although the evidence provides support to the importance of avoiding prolonged, uninterrupted periods of sitting for cardiovascular health in adults, further evidence from intervention trials is required.

Promotion of sitting begins at schools at the latest and increasing number of evidence show inactivity-related symptoms also in adolescents and children [16]. Interestingly, the amount of parent's sedentary time, nor their individual physical activity, seems to transfer to their children [17,18]. On the other hand, parent's and siblings' participation in physical activity with a child and reinforcement for physical activity seems to be important [19]. Thus, while our society supports sitting and motorizing every action, promoting physically active lifestyle as early as from childhood is crucial.

Based on Bandura's [20] social cognitive theory, a child learns by imitating and copying other people and by making own reasons founded on these social situations. Own parents and siblings act as one of the most powerful role models to a young child. Thus, the family environment is in key position influencing child's physical activity habits taking shape in the childhood [19,21-23].

The effective family interventions focused on increasing physical activity in children have prompted parents to set specific goals for changes in physical activity behaviour and to self-monitor behaviour change progress and to identify barriers faced. It has also been important that behaviour change techniques have spanned the spectrum of behaviour change process [24]. Parents have been shown to be motivated to their role as important models, if they receive knowledge why physical activity (PA) is important to their children. They also seem to profit from concrete ideas and model how to promote physical activity with their children [23].

For young children the parent's encouragement toward outdoor play seem to be essential [25] because the amount of outdoor play correlates with overall physical activity [26,27]. Further, playing outdoors and intensity level of moderate to vigorous physical activity (MVPA) correlates with fundamental motor skills (FMS) [28-30]. The more children are physically active the better FMS they have and the more possibilities they have to be physically active [31]. Good FMS during childhood predict physical activity and physical fitness during adolescence and later in adulthood [29,32,33]. On the other hand, poor motor skills or clumsiness predicts low physical activity and poor physical fitness [34].

The purpose of this study is to find out the effects of a tailored family based intervention on the activity level of both parents and their children. In parents with a sedentary job we investigate whether simple actions to reduce time in sitting position are viable and whether they have health-related benefits. Work-related inactivity is one important aspect of the present study, since in Finland 46% of women and 51% of men sit daily at least 6 h [35]. At the same time, we examine whether the counselling delivered to parents on the importance of children's physical activity transfers to the increase in children's physical activity level and hereby facilitates development of FMS.

The purpose of this article is to describe the design, setting, recruitment process, methodology and intervention of this randomized controlled trial.

## Methods

### Design

Parallel-group randomized controlled intervention trial (RCT) design is used with one intervention group and one control group of adults and their children. Adults in the intervention group receive tailored counselling to decrease time in sitting position and to increase non-exercise daily activity. This counselling is also targeted to encourage parents to increase their children's time spent outdoors and to increase children's intensity of activity toward MVPA level. Parents are also helped to recognize the amount of children's indoor activities, especially sedentary behaviour like TV viewing and playing video games.

An ethics approval for the project has been received from the Ethics committee of Central Finland Health Care District on March 25, 2011 (Dnro 6U/2011). Before signing consent, an info meeting is held where the subjects are informed about the procedures, risks and benefits of the study, and where they can ask questions. Subjects are volunteers with right to withdraw from the study at any time without consequences. Consent for the children to participate in the study will be signed by both legal guardians. The study is conducted according to Declaration of Helsinki and good scientific practice.

### Subjects

Eligible participants are parents with work that primarily includes sitting and their children of 3-8 years of age. *Inclusion criteria*: healthy men and women with children 3-8 years old, and having an occupation where they self-reportedly sit more than 50% of their work time. Children in all-day day-care in kindergarten at least 10 days per month or in the first grade in primary school. *Exclusion criteria*: self-reported chronic, long-term musculoskeletal disease or progressive neurological disease, diagnosed cardiovascular or metabolic disease with regular medication, families with pregnant mother at baseline and BMI > 35. Children with a developmental disorder or other disorders delaying motor development are excluded.

### Sample size

In the entire project we aim to see the main effect of the intervention as reduced inactivity time in adults. Sample size was calculated by assuming that the intervention could induce a decrease in the longest muscle inactivity periods from 6.37 ± 3.42 min to 5.22 ± 2.59 min in persons sitting 50-100% of their work time. This assumption was based on our data from previous project [36] where a mean duration of five longest continuous inactivity periods was measured from 84 people with self reported sitting (%) during work. A sample size of 86 in both groups would have 80% power to detect this 18% difference at the level of α = 0.05. Estimating a small cluster effect (1.133) [37] and drop outs a sample size of 100 in both groups was reached. However, since we also aim to show health effects of the intervention, we used changes in plasma triglycerides as an example of potential metabolic changes to estimate the power of the design with 100 subjects. Olsen et al. [38] reported triglycerides to change with 14 days reduction in ambulatory activities from 617 ± 56 μmol/l to 647 ± 47 μmol/l. With the assumption that this effect is reversible within a year with increased ambulatory activity, a sample size of 100 in both groups will have 98.4% power to detect this change. In addition, while conventional thinking holds that sample size is the primary adjustable variable for increasing statistical power, effect sizes depend on phenotypic definition and will likely increase the closer one connects to the underlying biochemistry [39]. Since the metabonomic analysis provides more detailed phenotype characterization this translates to even greater statistical power.

### Setting and randomization

The recruitment is performed in the city of Jyväskylä, Finland, by delivering advertisements to parents via kindergartens and primary schools which have been pre-randomized to control and intervention groups after balancing different environmental and socioeconomic regions within the city. Jyväskylä with 130,000 residents and surface area of 1171 km^2 ^has relatively small city centre and suburbs are characterized with proximity of lakes and forests. In the city opportunities for active commute using built-in bike paths and sidewalks are numerous although winter with snow creates challenges for a year-around commute. Also many of the kindergartens and primary schools have the possibility to utilize the natural environments in their daily program.

The compulsory education begins in August at the same year children have their seventh birthday. Before the first school year, 96% of Finnish children participate to a year-long voluntary preschool [40]. The average enrolment rate of children aged 3-5 years in childcare and early education services is 74% in Finland which is slightly lower than average of 77% in OECD-countries [41]. Kindergarten teachers have the pedagogical responsibility of children's early education and their proficiency requirement is 3-5-years of university or polytechnic education.

The recruitment started in April 2011 with five kindergartens that resulted in about 16% response rate. Based on this initial result we estimated that randomization of 32 kindergartens and schools would be required to reach required sample size. In the randomization the areas within the city centre and in different types of suburbs were balanced. Regarding socioeconomic regions, information from city registry was used to balance the areas. Thus, in each type of environment and socioeconomic region there were to be two or more kindergartens and/or primary schools that were then randomized into control and intervention groups.

### Intervention

Tailored counselling by research personnel (TF, AL, AP) is targeted to decrease time in sitting position and increase non-exercise activity during workdays (commute and work time) and in leisure time (evenings and weekends) in adults. A common 30-min lecture is given to maximum of six participants accompanied with face-to-face discussions. The lecture contains research-based justifications for physical activity recommendations, effects of physical activity and, specifically for adults, information why reduction in sitting time can be beneficial.

In the discussions the subjects will be first asked to describe their current way of commute to work and to think of feasible ways of increasing active modes of commute. Then goals set by the subject regarding the mode and frequency of the change (e.g. Instead of taking the bus I will bicycle to work *i*) sometimes, *ii*) once a week, *iii*) several days a week, *iv*) every day) will be marked down to a form.

Second, the work time will be discussed and the subjects are asked to identify items that can be modulated, e.g. standing in coffee breaks, walking about while talking to phone, breaking long periods of sitting, taking a walk before lunch etc. Third, considering leisure time (both weekdays and weekends) is especially focused to encourage families to spend time together in nature and non-built environments, playgrounds and parks where children can get diversified stimulation for play. In discussions the parents are also encouraged, if needed, to reinforce children to play outdoors with peers, to acquire motivational equipment for play, to maintain summertime physical activity level and to utilize the material given. Moreover, leisure time habits will be asked and changes in habits such as walking to grocery shop instead of driving by car or walking stairs instead of taking the lift are discussed.

The items that the subjects themselves suggest to modify will be written down and an agreement document will be signed by the subject and researcher to confirm the intended changes in behaviour.

During counselling, material for break exercises during work time, children's indoor and outdoor activities and local places for hiking are delivered. Documents "License to Move" and a fair copy of the agreement are given. The subjects are also informed about project web-page [42] containing motivational material for intervention group which will be regularly updated.

The counselling is reinforced by a phone call after 1 and 5 months during which the compliance is asked and modifications to the agreement can be made. During the phone discussions execution (both compliance and barriers) of each of the goals are asked and modified when required. In addition, motivational e-mails with information on health promotion by life-style physical activity for adults and illustrative tips to increase physical activity and play developing FMS in children are sent monthly. This reinforcement period lasts for 6 months. At Midline the subjects in the intervention group will be given individual feedback of their daily inactivity and activity times (from Baseline muscle activity measurements). Feedback of children's motor skills with motivational tips will also be given at Midline. After the Midline there will be no researcher contact with the subjects except for the 9 month measurements.

The control group will not receive the counselling intervention but will undergo the measurements similarly as the intervention group. After 12 month-measurements the control group will be invited to a feedback and counselling session where they have the possibility to receive the same information as the intervention group.

### Study protocol and outcomes

#### Protocol for adults

**Figure 1:**
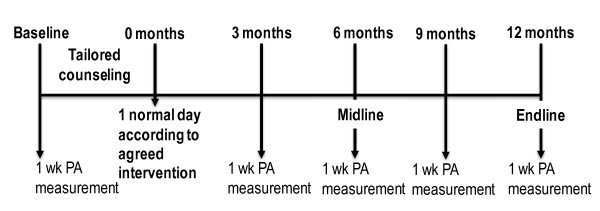
**Timeline of the project 1 week physical activity measurement (1 week PA measurement) refers to accelerometer measurements from both adults and children**. In adults also heart rate for 3 days and 2 nights with orthostatic test in the morning are assessed at these time points as well as fasting blood samples. At baseline, midline and endline questionnaires, body composition, daily activity tasks are assessed. At these 3 time points PA measurements include 1-day thigh muscle activity measurements.

The timeline of the study is shown in Figure 1. Baseline, Midline and Endline measurements contain fasting blood samples, anthropometry, questionnaires, strength measurements and functional tasks. These measurements initiate 1 week physical activity recordings during normal daily life. Counselling takes place few weeks after the baseline, and the maximum effect of counselling on physical activity will be tested about 1 week after the counselling (0 months). At this time data will also be collected from control group who is told that this assessment relates to reliability and they are to live a normal life similarly as in all the other measurements. The measurements at 3 and 9 months include only fasting blood sample, heart rate and accelerometer measurements.

**Figure 2:**
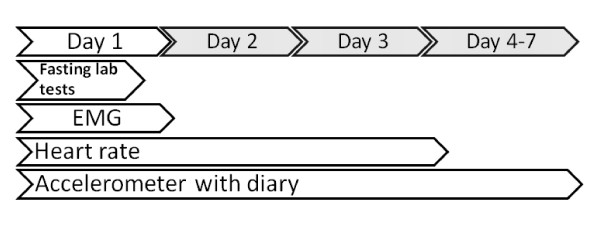
**Timeline of baseline measurements at baseline the fasting laboratory tests were followed by 1 day recording of muscle EMG activity, diurnal measurements of heart rate for 3 days and accelerometer measurements for 7 days**.

Baseline, Midline and Endline measurements initiate physical activity monitoring during normal daily life (Figure 2). The subjects will wear shorts measuring muscle activity for 1 day, heart rate device for 3 days and 2 nights and accelerometer for 7 days. During the days of physical activity data collection the subjects fill in physical activity diaries with information on sleep quality, alcohol usage and particularly stressful situations.

#### Outcome measures from adults

*Short term main outcome *is the PA behaviour which will be assessed with electromyography (EMG) and accelerometer. PA measured using EMG during 1 day at baseline will be compared to the daily measurements done after counselling. During this day, that is similar workday as in the baseline, the subjects are instructed to carefully execute the agreed changes in their PA behaviour allowing examination of maximal effect of the counselling. In the control group, this measurement will serve repeatability assessment. Commute time, work time and leisure time will be analyzed separately.

**Figure 3:**
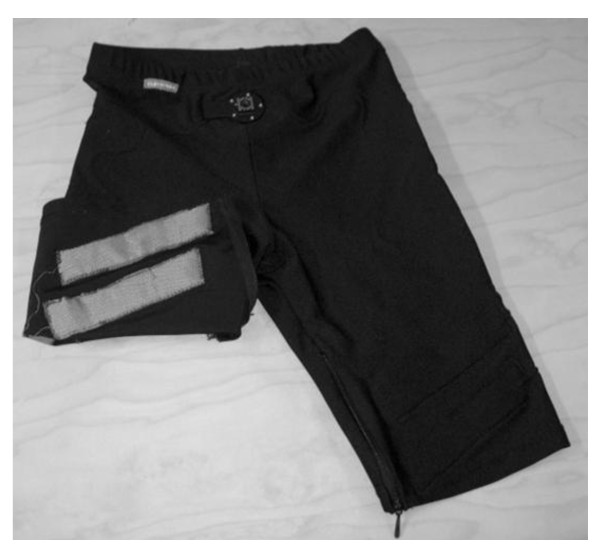
**EMG shorts used to collect muscle EMG activity from thigh muscles**.

*EMG *will be measured using shorts with embedded textile electrodes (Myontec Ltd., Kuopio, Finland) measuring activity from left and right quadriceps and hamstring muscles (Figure 3). The measurement system has been tested for validity, repeatability and feasibility in our laboratory [43]. The data is collected to a 52 g module in the waist in raw form with sampling frequency of 1000 Hz. The raw EMG signal is rectified and averaged over 100 ms non-overlapping intervals before storing the data into the module. The daily EMG level will be normalized to that during isometric maximum voluntary contraction. Inactivity threshold is individually determined as 0.9% of the standing EMG level. Several activity and inactivity parameters will be analyzed using custom made Matlab program including burst analysis [44].

##### Acceleration

Triaxial acceleration will be collected during all the measurements (Alive heart monitor, Alive technologies Ltd., Australia). We will analyze the commonly used activity counts but also use signal half power and histograms to describe changes in PA behaviour. Alive monitor can simultaneously collect also electrocardiogram from which the heart rate variability and related stress indices are calculated using Hyvinvointianalyysi-software (Firstbeattechnologies Ltd., Finland).

*Long term main outcomes *are health-related indices and maintenance of the behavioural change. Changes in physical activity behaviour will be assessed using EMG, accelerometer and questionnaires. The health indices to be collected 5 times a year are fasting blood samples and diurnal heart rate (Figure 1). Blood pressure and body composition with dual energy x-ray absorptiometry (DXA, LUNAR Prodigy, GE Healthcare) will be measured at Baseline, Midline and Endline in fasting condition.

*Venous blood samples *for whole blood count, blood lipids and glucose will be taken in standardized fasting conditions in the mornings 7-9 am. Samples will be analyzed using standard methods in clinical use. Serum will be stored at-80°C for later analysis of the entire serum metabolome via NMR spectroscopy. This methodology provides information on ~150 primary metabolic measures and ~100 derived variables with clear biochemical interpretation and significance. The directly measured metabolites include lipoprotein subclass distribution with 14 subclasses quantified including particle concentrations and individual subclass lipids, low-molecular-weight metabolites such as amino acids, ketone bodies, and creatinine, and detailed molecular information on serum lipid extracts including free and esterified cholesterol, sphingomyelin, degree of saturation and ω-3 fatty acids. Derived variables include selected ratios of metabolites implicated in lipolysis, proteolysis, ketogenesis and glycolysis as well as reagents and products of enzymatic reactions and measures obtained with the extended-Friedewald formula, eg. apolipoprotein A-I and B [45]. Further details of the NMR spectroscopy, quantification data analyses, as well as the full metabolite identifications can be found from [46,47].

*Heart rate *will be measured with the same device as acceleration. The subjects wear the monitors for 3 days and 2 nights. In the morning the subjects will perform an orthostatic test. Previously, heart rate variability from this test has been shown to correlate with self-reported stress [48]. Both time domain and frequency domain analysis of heart rate variability will be performed, where high frequency band has special meaning in determining cardiovascular health [49].

##### Questionnaires

Background information of medication, chronic and acute diseases will be assessed in the screening process. Dietary records for 3 weekdays and 1 weekend day will be kept at Baseline and Endline and 1 day diaries at 3, 6 and 9 months to evaluate whether similar diet has been maintained during the year as assumed. The subjects will also fill in the following questionnaires: Socio-economic background (Baseline), 12-month physical activity (Baseline, Endline, modified from [50], quality of life (RAND-36, Baseline, Endline), Work Ability Index (Baseline, Endline) and Occupational Stress Questionnaire (Baseline, Midline, Endline) (Finnish Institute of Occupational Health). Physical fitness will be assessed with non-exercise questionnaire (Baseline, Endline) and additional details of physical activity intensity and frequency will be asked at all time points. Family-Influence Model questionnaire [19] will be assessed in Baseline, Midline and Endline.

### Protocol for children

At Baseline, 3, 6, 9 and 12 months, children will be measured for physical activity level during waking hours for 6 days. During the days of physical activity measurements the parents will mark down to the children's diary the times the accelerometer were put on and taken off. In addition, parents are asked to subjectively estimate the amount of children's MVPA time after day-care or school. At Baseline, Midline and Endline children will also be measured for FMS.

### Outcome measures from children

Physical activity will be measured using triaxial accelerometer (X6-1A USB Accelerometer, Gulf Coast Data Concepts, USA) which is placed on children's waist with firmly fitted elastic band. The time at day-care or school will be analyzed separately from leisure time. Extracted variables will include commonly used activity counts based on thresholds [51], histograms and half power of the signal. FMS will be tested using Körperkoordinations Test fur Kinder (KTK) [52] for balance and motor coordination and using APM object control test [53]. APM is a widely used motor skill test for children in Finland. FMS tests are conducted as part of daily activities in kindergartens, schools and, in some cases, in the laboratory of Department of Biology of Physical Activity.

### Statistical analysis

The maximal effect of counselling will be tested using mixed model ANOVA comparing the control and intervention groups at baseline and at 0 months. Repeated measures ANOVA will be used to determine effects of intervention with an intention-to-treat principle. Mixed models are used in case of missing data points and to examine the clustering effect as well as to examine the relationships between physical activity and health-related indices. Self-organizing maps (SOM) will be applied for multi-metabolic understanding of metabolic interrelationships and pathways [54,55].

## Discussion

This study is unique in implementing intervention to both parents and their children at the same time. The intervention focusing on reducing inactivity rather than increasing physical activity is a rather new concept and the actual execution by the adults is likely to determine the significance of this study. For the intervention group we have carefully designed a motivating environment with face-to-face discussions where the goals will be set by the subjects themselves and the commitment is formalized by signing an agreement. This type of intervention has been shown to be very effective [56].

To reliably extract the possible changes in physical activity the present study utilizes both conventional (such as activity counts from accelerometer) and novel methods (histograms and signal half power from accelerometer and EMG) for more accurate determination of physical activity intensity and durations. Further, the measurements are done long enough periods 5 times during the year to assess the altered behaviour. Whether the altered behaviour in adults is reflected to health, it is postulated that the metabonomics analysis is sensitive enough to detect even subtle changes that would not be visible in conventional clinical markers.

There are challenges in this trial. Firstly, the compliance cannot be foreseen. The subject group is at very busy stage of life making careers with small children and a year-long commitment may result in considerable attrition rates. Further, seasonal variations in Finland with warm summer and cold winter with snow may show a greater effect on physical activity and health indexes than the low threshold intervention. We have considered this effect by performing the baseline measurements sequentially in spring and fall seasons.

Overall, this study has the potential to provide novel and timely information on the cause-effect relationships of low threshold physical activity intervention. However, the effectiveness of the intervention can only be assessed after the end of the study.

## Abbreviations

ECG: Electrocardiogram; EMG: Electromyography; FMS: Fundamental motor skills; MVPA: Moderate-to-vigorous physical activity; NMR: Nuclear magnetic resonance; PA: Physical activity.

## Competing interests

The authors declare that they have no competing interests.

## Authors' contributions

TF, AS and SS initially designed the study and obtained funding for the research. All authors contributed to developing the protocols and intervention materials. TF, AL and AP drafted the manuscript and all authors were involved in revising it for intellectual content and have given final approval of the version to be published. All authors read and approved the final manuscript.

## Pre-publication history

The pre-publication history for this paper can be accessed here:

http://www.biomedcentral.com/1471-2458/11/944/prepub
